# Preventing Nasal Alar Necrosis in Oral Cancer Patients: A Quality Improvement Initiative for Safer Nasotracheal Intubation at a Tertiary Cancer Centre

**DOI:** 10.1007/s13193-025-02422-5

**Published:** 2025-09-15

**Authors:** Shivakumar Thiagarajan, Rukmini Prabhu, Vandana Agarwal, Vijaya Patil, Chandrashekar Dravid, Sarbani Ghosh-Laskar, Gouri Pantvaidya

**Affiliations:** 1 Division of Head & Neck, Department of Surgical Oncology, Tata Memorial Centre and Homi Bhabha National Institute (HBNI), Mumbai, India; 2 Department of Anaesthesiology, Critical Care & Pain, Tata Memorial Centre and Homi Bhabha National Institute (HBNI), Mumbai, India; 3 Department of Radiation Oncology, Tata Memorial Centre and Homi Bhabha National Institute (HBNI), Mumbai, India

**Keywords:** Nasal alar necrosis, Head and neck cancer, Surgery, Nasotracheal intubation

## Abstract

Nasal alar necrosis (NAN) is a known but easily avoidable complication of nasal intubation in patients undergoing head And neck cancer surgery. It is preventable, And the presence of NAN can be considered a poor quality of service rendered to the patients. This study was done as part of the quality control aspect in the institution. The aim was to sensitise the stakeholders to the existence And identification of this problem, identify factors predisposed to the development of NAN, And act on them to reduce its incidence in the hospital. A total of 141 patients were operated on between 1st Oct 2023 And 31st Dec 2023, of which 77 patients had nasotracheal intubation. The incidence of NAN was seen in 28 patients (36.4%). The factor predisposing to the development of NAN was overnight intubation (*p* < 0.001). Twenty-seven out of the 28 patients with overnight intubation developed AnN. Various key drivers were identified, And interventions were introduced in January. The incidence of NAN was subsequently recorded fortnightly, like before, till May 15th 2024. The incidence reduced over time to 7% from the initial 36.4%. NAN is a preventable postoperative complication in patients undergoing head and neck surgery with nasotracheal intubation. Constant effort is needed to monitor its incidence and sustain the factors implemented to prevent this complication.

**Supplementary Information:**

The online version contains supplementary material available at 10.1007/s13193-025-02422-5.

## Introduction

**Fig. 1:**
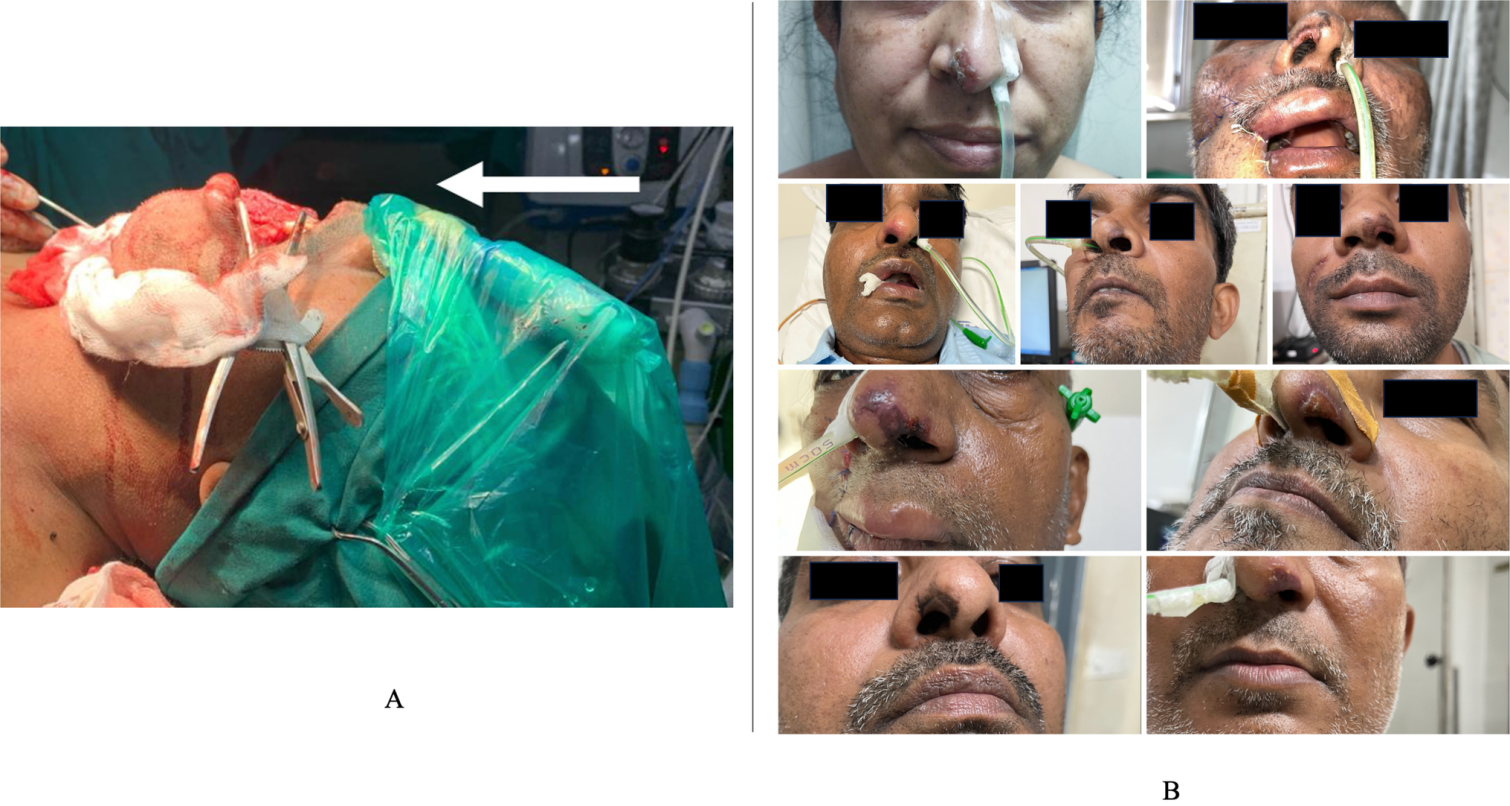
(**A**) Nasotracheal intubation (NTT) connected to the anaesthesia ventilating equipment, (**B**) Nasal Alar Pressure Sores (NAPS)

Head and neck surgeries are complex, necessitating resections and reconstruction, often lasting for several hours. Often, these patients are operated on with nasotracheal intubation (NTT). The NTT is connected to the anaesthesia ventilating equipment via tubes/circuits, which can give rise to a constant drag on NTT and, consequently, on the nose/nostril (Fig. 1A). This in turn can give rise to pressure on the nostril/nasal ala and cause pressure sores called nasal ala pressure sores (NAPS) (Fig. 1B). These are usually minor and easily ignored. NAPS may also get infected and pose a risk, given their location in a dangerous area of the face. They also give rise to an obvious scar, adding to the disfigurement of these patients.

Nasal alar necrosis is a known, easily avoidable, and often neglected complication of NTT. It was first reported by Hatcher et al. in 1968 [1]. Most head and neck surgeries require frequent changes of head position and overnight intubation. As the NTT is close to the surgical site during head and neck surgery, there is also a tendency for surgeons to inadvertently put pressure on the catheter mount connecting the NTT to the anaesthesia machine by leaning on it. Another possibility is the fixation of NTT at the nostril. The constant pressure on the ala nasi causes a decrease in the capillary blood supply and, subsequently, NAPS [2]. The incidence of alar necrosis post-head And neck surgery has been reported to be between 25 And 51.4% [3–5]. The nose, especially the nasal alar, is devoid of a fat cushion and hence prone to injury in the presence of constant pressure. The risk factors that may be associated with ala nasi pressure sores are gender, prolonged duration of surgery, type and material of endotracheal tubes, bare contact surface, and sharp angle between the tube and nose [6, 7]. The effects of nasal alar necrosis may be minor and self-limiting, but in some cases, there might be severe necrosis with significant cosmetic and functional issues and, hence, poor satisfaction [2]. This is a preventable postoperative complication, and the presence of NAPS could be considered a suboptimal quality of service rendered to the patients. Hence, in this study, we aimed to determine the incidence of alar necrosis in patients undergoing head and neck surgery, risk factors associated with alar necrosis, and suggest remedial measures to prevent/decrease the incidence of NAPS at our institute.

## Methodology

A core multidisciplinary team of Surgeons, Anaesthetists, and Nurses participated in the ‘Enable Quality Improve Patient Care (EQuIP)’, a Stanford-India Collaborative Quality Improvement (QI) training project from 2023 to 2024. Other team members included the Quality Training Program mentors (two institutional). This study was undertaken as a QI initiative at Tata Memorial Centre, Mumbai, rather than human participant research. The study was conducted according to the Declaration of Helsinki, as revised in 2013. Institutional Ethics Committee approval has been obtained for this study.

The problem statement for the project was the lack of identification of NAPS as a problem by the stakeholders (Surgeons, Anaesthetists and Nurses). We also did not have the actual incidence of NAPS at our institute before initiating this study and, subsequently, the lack of understanding of probable predisposing factors. We started with setting two SMART (Specific, Measurable, Achievable, Relevant, and Time-Bound) goals: firstly, to understand the incidence of NAPS, sensitise the existence of this problem to stakeholders, And consider this as a measure of the quality of care delivered to our patients. Secondly, we wanted to identify predisposing factors for the development of NAPS And reduce the overall incidence of NAPS to less than 10% of its current incidence (once identified) in 3 months. The team met fortnightly during the project and attended online lectures on various steps of QI.

### Part 1 (Cohort 1)

To achieve the first SMART goal (to achieve baseline data), all patients aged > 18 years undergoing surgery for oral cancer with nasotracheal intubation from October 1, 2023, to May 15, 2024, were included. Patients undergoing surgery with oral intubation/tracheostomy for oral cancer or other head and neck cancer were excluded. The relevant demographic and clinical details such as age, gender, comorbidities, ECOG status, and history of prior treatment (including head and neck irradiation), family history, clinical T-stage, N-stage, details of the surgery, duration of surgery, need for overnight intubation, duration of intubation, and location of postoperative ward were collected from the electronic medical records. The patient was assessed on the first postoperative day by the surgeon (Consultant & Resident) for the presence or absence of NAPS. Any sore or ulcer on the nasal ala was documented as the presence of NAPS (Fig. 1B). Baseline demographics And relevant clinical information are documented for periodic clinical audit. Patients with NAPS were treated with topical Antibiotic ointment for local application for 7 to 10 days.

### Statistical Analysis

Statistical Analysis was done using SPSS version 29 (IBM Corp, Armonk, New York). The univariate analysis was done to test the association for variables based on clinical relevance using the chi-square test and/or Fisher’s exact test. The multivariate analysis was done using binomial logistic regression (forward stepwise selection). A *p*-value of < 0.05 was considered significant.

### Part 2 (Cohort 2)

**Fig. 2:**
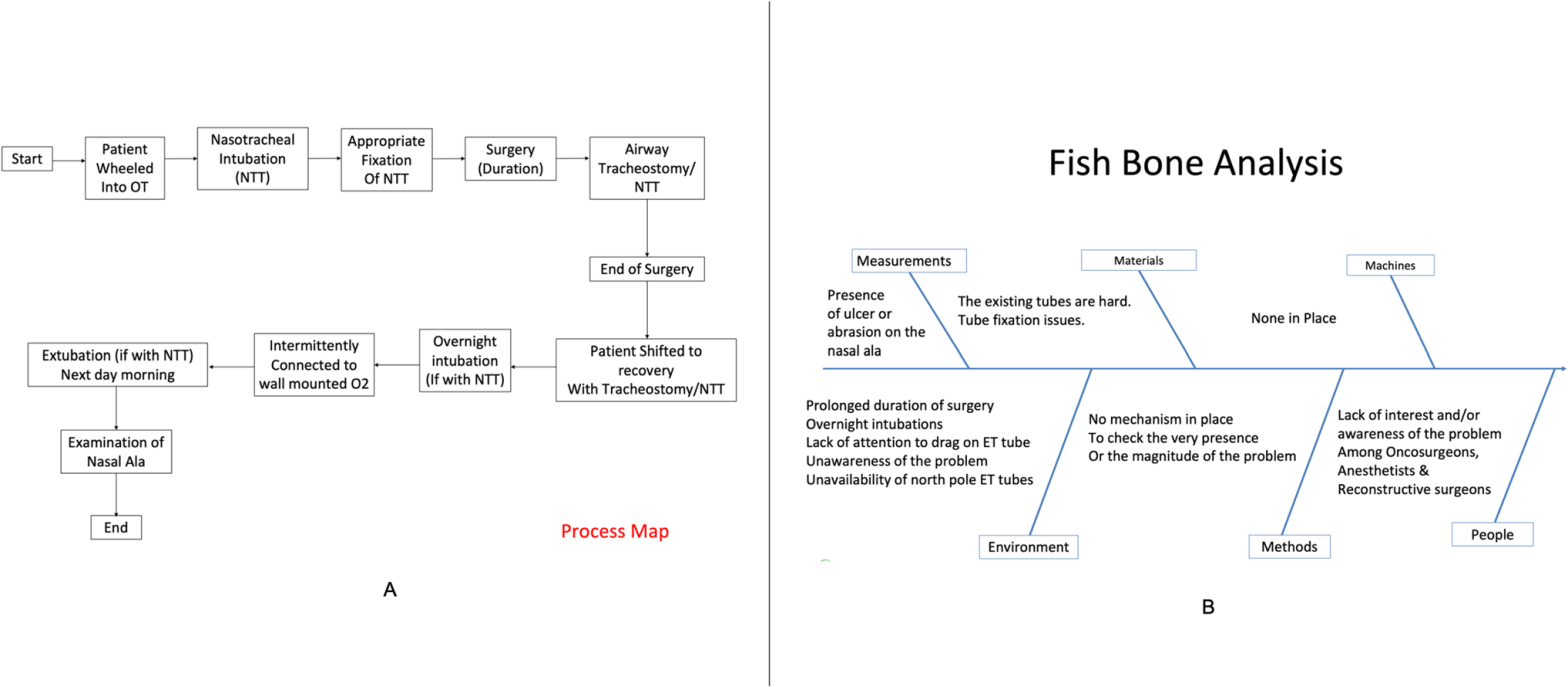
(**A**) Process map, (**B**) Fish Bone Analysis

**Fig. 3:**
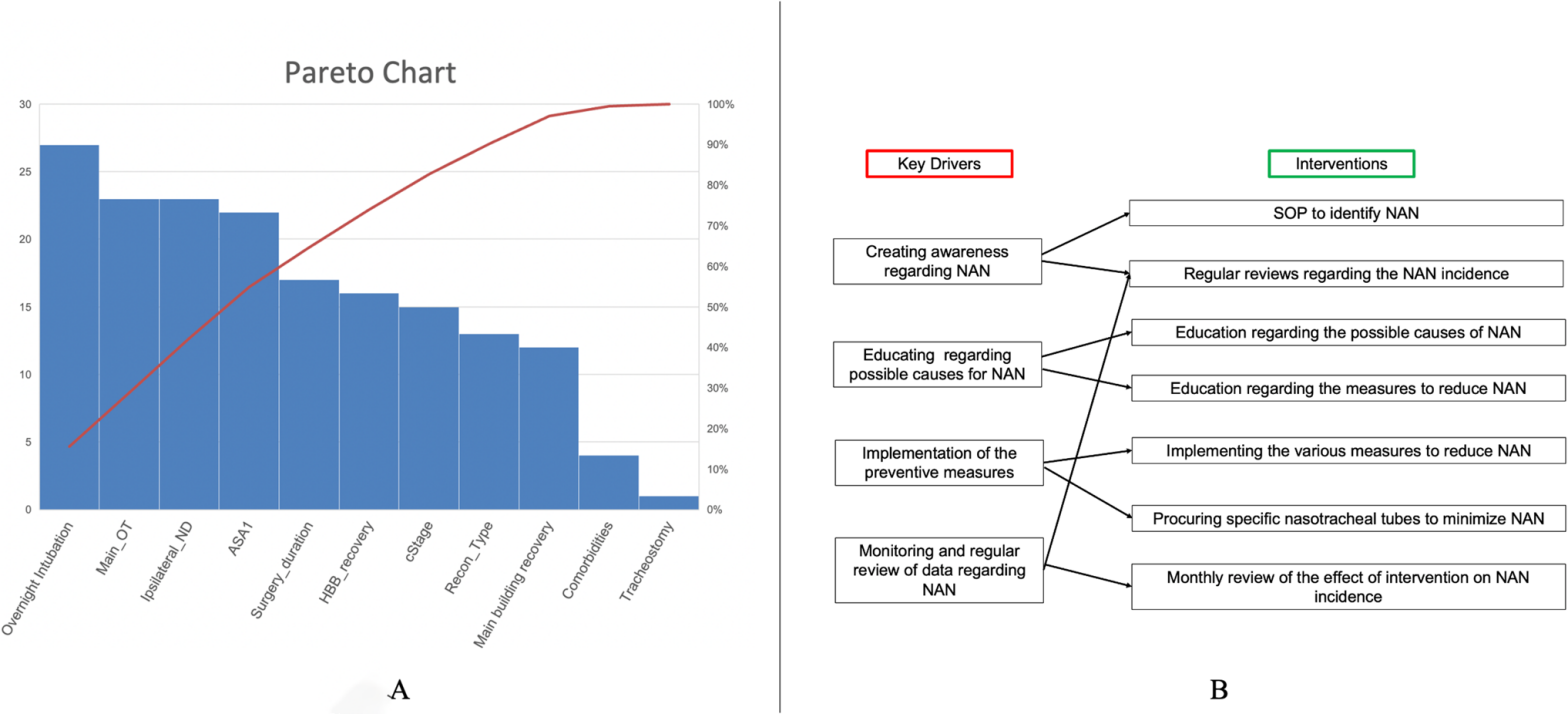
(**A**) Pareto chart, (**B**) Key drivers and interventions

Information regarding the incidence and predisposing factors was shared with all stakeholders. Several meetings were held with various disciplines involved in the clinical care pathway to generate and review the process map and fishbone diagram. A process map (Fig. 2A) was made to understand the sequence of events starting with patients entering the operating room and until shifted to the postoperative recovery bay. Next, a fishbone analysis was done to identify potential contributory factors for the development of NAPS (Fig. 2B). As a team, the stakeholders were asked to prioritise the most frequent causes for NAPS; this was used to generate a Pareto chart (Fig. 3A). Interventions were designed to address these areas that were easiest to implement and provided maximum benefit to patients and the care provider team (Action-Priority-Matrix).

**Fig. 4:**
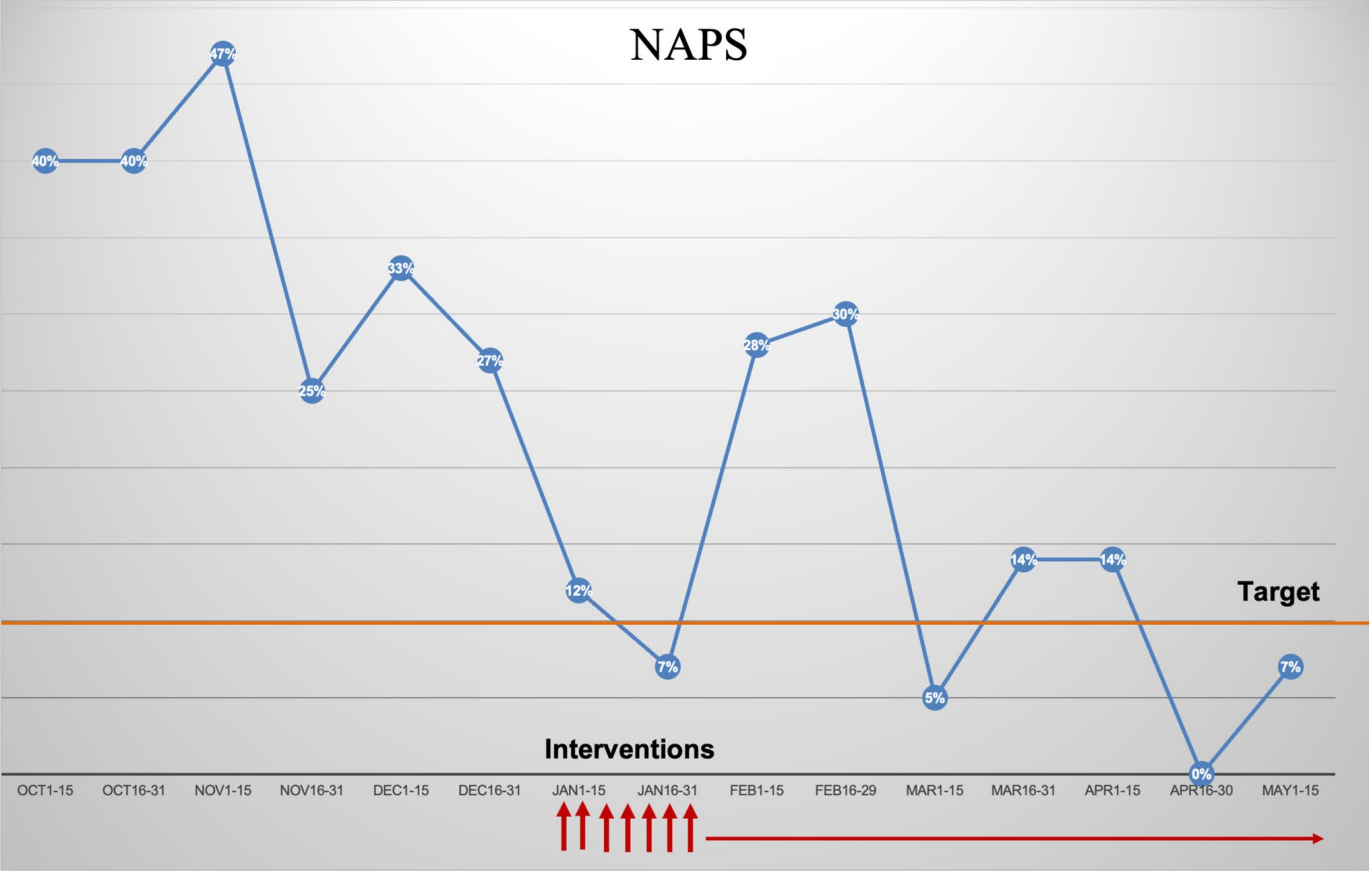
Run Chart showing the incidence of NAPS across the study point along with the time point where interventions were implemented (Arrows) and the subsequent reduction in its incidence

Plan-Do-Study-Act Cycle (PDSAC): It was perceived that there was a lack of understanding/awareness of the entity NAPS as a complication following nasotracheal intubation for oral cancer surgery. Also, there were no preventive measures/methods to monitor NAPS. Hence, a set of interventions was conceived and implemented (Fig. 3B). These interventions were implemented in January 2024. The incidence of NAPS was monitored fortnightly during the implementation process of various interventions (Fig. 4, red arrows) and subsequently thereafter as well.

## Results

### Part 1 (Cohort 1)

Between 1 st October 2023 And 31st December 2023, a total of 141 cases underwent head And neck surgery, 77 (54.6%) of these patients were intubated with nasotracheal tubes for oral cancer surgery, fulfilling the eligibility criteria And therefore included in the Analysis. Overall, NAPS was seen in 28 of 77 patients (36.4%). The incidence of NAPS was plotted on a fortnightly basis to capture the trend over time (Fig. 4). Patients requiring overnight intubation (*p* < 0.001) appeared to be the most common predisposing factor for the development of NAPS. In addition, the method of NTT fixation was considered one of the contributing factors. Other predisposing factors for the development of NAPS are mentioned in the Pareto chart (Fig. 3A). Our smart goal was to reduce the incidence of NAPS to below 10%. A process map was created (Fig. 2A) to understand the workflow. Next, the fishbone analysis (Fig. 2B) and Pareto chart (Fig. 3A) were generated to understand various reasons for NAPS.

### Part 2 (Cohort 2)

Key drivers for the development of NAPS were generated based on the above findings, and interventions to neutralise them were also formulated (Fig. 3B). These were reinforced to all stakeholders. Some aspects of key interventions were to educate the stakeholders regarding the handling of NTT tube, including proper fixation before the surgery begins and constant vigilance of the NTT position throughout the surgery (Fig. 5, Supplementary), use of catheter mount in all head And neck cases, And avoidance of drag on the NTT. The NTT should be fixed in a way that the drag on it and consequently on the nasal ala is eliminated as shown in Fig. 5 (Supplementary). The NTT should be fixed on the operating table as shown in Fig. 5 (Supplementary) rather than allowing it to hang on its own weight. A policy to minimise overnight intubation as much as possible was undertaken. However, in cases where overnight intubation was necessary, certain measures were taken, such as making the ward nurse aware about the condition of NAPS and making sure that when the wall-mounted oxygen is connected to the NTT, there is no drag on it and that the ward nurse could intermittently have a check on the same overnight and correct if there is a drag on the ETT to prevent NAPS, when in patients requiring overnight intubation, refixation of the NTT, if necessary, and ensuring avoidance of drag in the postoperative ward while the patients were connected to wall-mounted O_2_ supply was emphasised. All the information concerning the incidence of NAPS and the key drivers/interventions was reinforced to the stakeholders at regular time intervals (Fig. 4). A total of 135 patients underwent surgery for oral cancer between 1 st January 2024 And 15th May 2024. The interventions were implemented in January 2024. The incidence of NAPS was plotted every fortnightly as mentioned earlier (Fig. 4), till 15th May 2024 in cohort 2 patients. Hence, after the introduction of the various interventions, over 3 months, the incidence of NAPS dropped to 7% from the initial 36.4%, thus achieving our smart goal.

To sustain the improvement that was achieved, a sustenance plan was implemented. For this, the authors of this article decided to document the presence of NAPS as one of the postoperative morbidities in the existing department database and audit every fortnight henceforth. The incidence of NAPS thus documented was shared with all stakeholders at regular intervals, and in addition, the measures needed to minimise this complication, as discussed earlier, were also reinforced to all at regular time intervals.

## Discussion

Following oral cancer surgery with nasotracheal intubation, NAPS was identified as a factor for suboptimal delivery of quality of care. The incidence of NAPS in our institute was 36.4%; thus, the goal was to reduce it to < 10% over 3 months. Prolonged duration of surgery, securing of NTT, and nasotracheal intubation (overnight) emerged as important causes for the development of NAPS. We introduced various interventions to minimise or avoid NAPS (such as preventing overnight intubation and changing the securing technique on the nose to prevent a cephalad drag on the nasal ala, and Surgeons were instructed to be cognizant of the nasotracheal tube position during surgery). These interventions reduced the incidence of NAPS from 36.4% to 7% in 3 months.

The postoperative complication of NAPS is easily missed often due to a lack of awareness and oversight; hence, there is limited literature. Studies have shown that constant pressure on soft tissue over a certain period may result in pressure sores [8]. Daniel RK et al. demonstrated that a small amount of constant pressure on soft tissue maintained for a long duration could give rise to tissue damage [9]. Similarly, in the present study, the long duration of surgery and prolonged overnight nasotracheal intubation were identified as factors associated with NAPS.

Sumphaongern T. [2], in a prospective study of 155 patients over the 6-month duration, reported An incidence of 21.45%. The long duration of surgery and lack of hydrocolloid dressing were reported as significant risk factors for the development of ala nasi pressure sores. A significant protective factor reported in the study was a higher body mass index (BMI). Shortening the duration of surgery and using hydrocolloid dressing between the ala nasi and NTT or catheters was recommended [2]. Ravindra T et al. suggested various methods, such as having an additional catheter mount to the NTT to minimise or avoid NAPS [10]. Huang et al. in their animal study reported use of soft materials such as Soft Liner and DuoDERM reduced the size and severity of NAPS [11]. Iwai T had reported the use of hydrocolloid dressing to prevent the development of alar nasal pressure sores following nasotracheal intubation [12]. Singh R et al. used Merocel ® in 33 patients during fixation of NTT for the prevention of NAPS [5]. Hashimoto et al. conducted a randomised trial using 3 M microfoam™ surgical tape versus hydrocolloid dressing And found that 3M microfoam™ was more effective in preventing the ala nasi pressure sore [13]. Anand R et al. reported the use of polyvinyl acetyl sponge packs to prevent NAPS [14]. Weng MH compared protective dressing, such as hydrocolloid dressing or film dressing, and no dressing in ventilated patients with NTT and reported that the use of protective dressing reduced the incidence and duration of NAPS [15].

Huang TT et al. suggested using long soft flexible, and lubricated NTT or armoured flexometallic tube for long-duration surgery, protective dressing between NTT and the nasal ala, and lastly paying attention while moving the patient’s head during surgery as preventive measures for NAPS [16]. While it is intuitive to consider using a north pole nasotracheal tube as a preventive measure, this is not available in our institution due to logistics issues (supply chain).

In our study, we have shown how clinical problems/quality of care issues can be addressed systematically and can be minimised or solved. The present study is very relevant as it has identified a quality of care delivery issue and circumvented it by locally available resources with education, the need for vigilance, and necessary intervention at no additional cost. However, the main challenge is to improve further with zero incidences and, at the very least, sustain these results.

## Conclusions

In this study, the incidence of NAPS was 36.4% in our institute. The method of NTT securing, the need for overnight intubations, And long-duration surgery were identified as factors contributing to patients developing NAPS. Various simple interventions were implemented, leading to a reduction in the incidence of NAPS to 7% in 3-month duration. We thus improve the quality of care delivered to our patients. We are currently following the same protocol to sustain the drop in NAPS that we achieved and eventually reduce it to zero. Also, we look forward to implementing this methodology in any area where we wish to improve the quality of care or service.

## Supplementary Information

Below is the link to the electronic supplementary material.

Supplementary Material 1 (JPG 71.5 KB)

## Data Availability

Due to ethical concerns regarding patient confidentiality, the data are not publicly available but may be shared upon request with appropriate institutional approvals.
