# Supplementary figures and images for: Preventing Nasal Alar Necrosis in Oral Cancer Patients: A Quality Improvement Initiative for Safer Nasotracheal Intubation at a Tertiary Cancer Centre
Source: Indian J Surg Oncol. 2025 Sep 15;17(6):1223–9. doi: 10.1007/s13193-025-02422-5 (PMC13315066; doi:10.1007/s13193-025-02422-5)

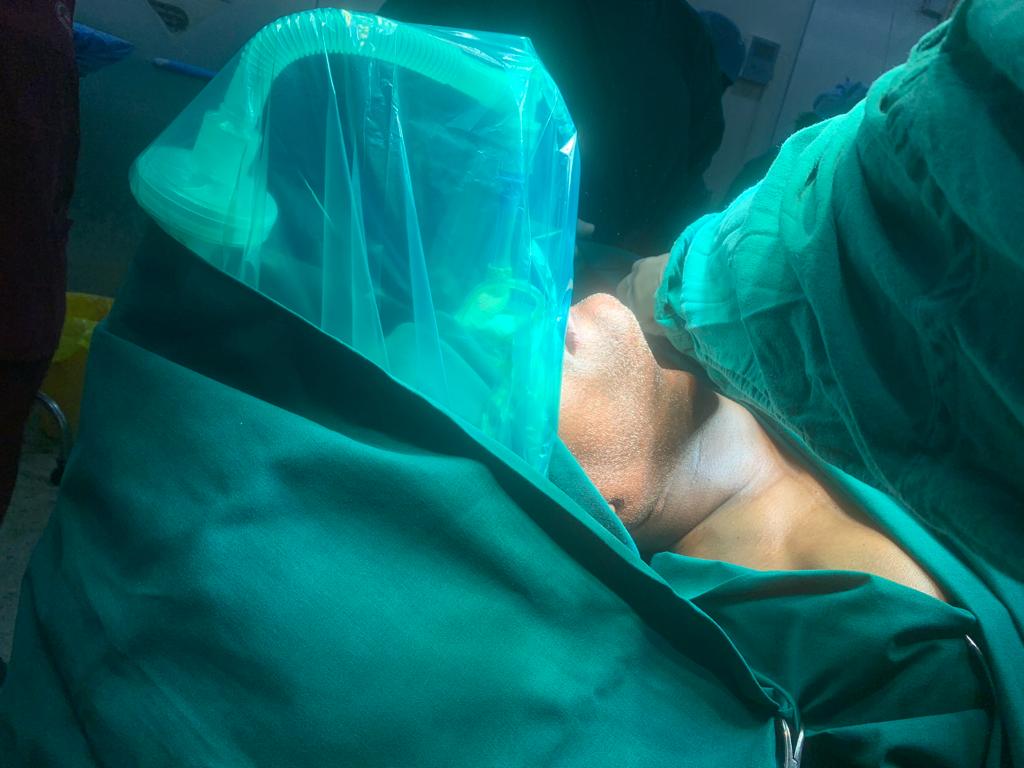

Supplement: Supplementary file 1 — Supplementary Material 1 (JPG 71.5 KB) [file 13193_2025_2422_MOESM1_ESM.jpg]
